# 奥希替尼在非小细胞肺癌靶向治疗中的获得性耐药机制

**DOI:** 10.3779/j.issn.1009-3419.2020.103.02

**Published:** 2020-04-20

**Authors:** 梓彤 赵, 羽 倪, 里 李, 涛 信

**Affiliations:** 150081 哈尔滨，哈尔滨医科大学附属第二医院肿瘤内科 Department of Oncology, The Second Affiliated Hospital of Harbin Medical University, Harbin 150081, China

**Keywords:** 肺肿瘤, EGFR, EGFR-TKI, 奥希替尼, T790M, Lung neoplasms, EGFR, EGFR-TKI, Osimertinib, T790M

## Abstract

表皮生长因子受体酪氨酸激酶抑制剂（epidermal growth factor receptor-tyrosine kinase inhibitor, EGFR-TKI）在治疗癌症的同时仍面临不可避免的耐药。通过研究EGFR-TKI发生耐药的机制从而发现一些新的分子标志物和药物靶点，促进了第三代TKIs的发展并针对耐药提出合理化建议。经临床验证，T790M是一个可用于判断预后的生物学标志物，可导致第一代和第二代TKIs的难治性。对于T790M阴性的患者，尽管靶向治疗和检查点阻断治疗结合可能提供有希望的替代方案，细胞毒性药物序贯EGFR-TKI治疗仍是疾病进展后可接受的标准治疗方式。在T790M阳性患者中，第三代EGFR-TKI药物奥希替尼在随机临床试验中优于铂类二联化疗和第一代EGFR-TKI。文章综述了近年来有关奥希替尼在非小细胞肺癌患者获得性耐药机制及治疗的主要文献，并展望了未来可能的研究方向。

## Abstract (en)

While treating cancer, epidermal growth factor receptor-tyrosine kinase inhibitor (EGFR-TKI) still faces inevitable drug resistance. Investigations into the mechanisms which foster resistance to EGFR-TKI has led to the discovery of novel biomarkers and drug targets, and in turn has enabled the development of third-generation TKIs and proposals for rational therapeutic combinations. The threonine-to-methionine substitution mutation at position 790 (T790M) is clinically validated to engender refractoriness to first- and second-generation TKI, and is a standard-of-care predictive biomarker used in therapeutic stratification. For patients who are T790M-negative, cytotoxic chemotherapy or protracted EGFR-TKI treatment are acceptable treatment standards after disease progression, although combinations of targeted therapies and checkpoint blockade immunotherapy may offer promising alternatives in the future. Among T790M-positive patients, the third-generation EGFR-TKI, osimertinib, has shown superiority over both platinum-doublet chemotherapy and first-generation EGFR-TKI in randomized clinical trials. This article appraises the key literature on the contemporary management of non-small cell lung cancer patients with acquired resistance to EGFR-TKIs, and envisions future directions in translational and clinical research.

非小细胞肺癌（non-small cell lung cancer, NSCLC）分子检测已经发现了一个多样化的并且仍在扩大的驱动和维持肿瘤发生的异常遗传基因目录。其中，表皮生长因子受体（epidermal growth factor receptor, *EGFR*）基因突变是肺癌致瘤驱动因素的典型例子，可以作为治疗的靶点。大量的随机试验和*meta*分析证实了小分子EGFR酪氨酸激酶抑制剂（tyrosine kinase inhibitor, TKI）对*EGFR*突变型NSCLC患者的治疗优于细胞毒性药物，在客观缓解率（objective response rate, ORR）和无生存进展期（progression-free survival, PFS）方面具有显著的优势^[1]^。

尽管有相当大比例患者最初使用EGFR-TKIs治疗取得了较好的疾病控制，病情进展仍不可避免^[2]^。考虑到肿瘤的形成和肿瘤持续进展对EGFR阻断序列的抵抗，EGFR靶向治疗产生耐药性的NSCLC患者的管理是一个独特且持续的挑战。奥希替尼作为第三代EGFR-TKI作用于EGFR激活和T790M耐药突变，同时对野生型EGFR的抑制作用很低^[3]^。本文综述了奥希替尼针对于*EGFR*突变型NSCLC的基础生物学和治疗方案，总结了第三代EGFR-TKI获得性耐药的病理生物学机制，并对EGFR-TKI的临床管理提出了当前的挑战和展望。

## 1 奥希替尼的药代动力学特点

奥希替尼（AZD9291）是口服不可逆的嘧啶基EGFR-TKI，通过半胱氨酸-797残基与EGFR T790M或*EGFR*突变形成不可逆的共价键，对*EGFR*敏感活化突变和T790M耐药突变具有选择性抑制作用^[4]^。在EGFR重组酶测定中，奥希替尼对L858R和L858R/T790M有显著的活性，半数抑制浓度（half maximal inhibitory concentration, IC_50_）分别为12 nmol/L和1 nmol/L，而对野生型EGFR的活性较低。在细胞抑制试验中也发现了类似的作用。通过奥希替尼对突变和野生型EGFR细胞的EGFR磷酸化体外活性发现，奥希替尼在抑制定位于*EGFR*敏感突变（ex19del和L858R）的肿瘤细胞中的EGFR磷酸化方面的作用与早期EGFR-TKIs相似，其IC_50_为54 nmol/L。在T790M突变细胞系中，奥希替尼在IC_50_低于15 nmol/L的情况下具有高效力。与早期EGFR-TKIs相比，奥希替尼在野生型细胞系中的活性较低。在人体中，奥希替尼的平均半衰期为48.3 h，达到血浆药物峰浓度（maximum concentration, Cmax）的时间为6 h，15 d后达到稳定状态。最大血药浓度呈剂量依赖性且呈线性药代动力学特点^[5]^。奥希替尼的主要代谢途径是氧化和脱烷基化，生成两种活性代谢物：AZ7550和AZ5104。与禁食状态下相比，进食高脂肪、高热量食物时，Cmax和药时曲线下面积（area under curve, AUC）分别增加14%和19%^[5]^。

## 2 奥希替尼的临床应用

AURA研究是对奥希替尼的Ⅰ期/Ⅱ期开放性多中心研究，研究对象为初期应用EGFR-TKI进展的晚期*EGFR*突变NSCLC患者。研究目的包括安全性、耐受性和有效性。在升级阶段，患者（*n*=31）没有通过T790M状态进行预选，而在扩展阶段，患者要么通过本地测试，然后通过中心实验室确认T790M状态，要么仅通过中心实验室测试进行登记。在升级阶段，共有157例患者被纳入5个剂量组（每日20 mg、40 mg、80 mg、160 mg和240 mg），在任何剂量组中都没有遇到剂量限制毒性。Ⅱ期临床试验推荐的剂量为每日80 mg且未达到最大耐受剂量。奥希替尼治疗T790M阳性NSCLC患者的客观有效率（objective response rate, ORR）为61%，且各剂量的ORR水平相似，ORR为50%-70%，疾病控制率（disease control rate, DCR）为95%，PFS为9.6个月。在T790M阴性NSCLC患者中，奥希替尼的活性较低，ORR 21%，DCR 61%，PFS为2.8个月（表 1）。

**1:** 选择性奥希替尼研究的临床结果 Efficacy outcomes in selected osimertinib studies

Study	Ref.	Phase	Treatment	ORR（%）			DCR (%)			PFS (mon)	
				T790M+	T790M-		T790M+	T790M-		T790M+	T790M-
AURA	Janne	Ⅰ/Ⅱ	Osimertinib	61	21		95	61		9.6	2.8
AURA extension	Yang	Ⅱ	Osimertinib	62	NA		90	NA		12.3	NA
AURA 2	Goss	Ⅱ	Osimertinib	70	NA		92	NA		9.9	NA
AURA 3	Mok	Ⅲ	Osimertinib or platinum-pemetrexed +/-maintenance pemetrexed	71 31	NA NA		93	NA		10.1 4.4	NA NA
DCR: disease control rate; NA: not applicable; ORR: objective response rate; PFS: progression-free survival; TKI: tyrosine kinase inhibitor.											

AURA试验的Ⅱ期扩展研究中，201例患者每天服用80 mg的奥希替尼，结果ORR为62%，DCR为90%，PFS为12.3个月^[6]^。AURA2是*EGFR*突变型NSCLC，且在EGFR-TKI使用后有进展，并证实为T790M患者使用奥希替尼的Ⅱ期单臂研究，其ORR结果为70%，DCR为92%，PFS为9.9个月^[7]^。一项Ⅲ期验证性研究——AURA 3，在未经化疗的EGFR-TKI治疗后伴有T790M突变的NSCLC患者中进行。共有419例患者被随机分配到奥希替尼或化疗组（培美曲塞+铂类），分配比例为2:1，后续加或不加培美曲塞维持治疗。最终的结果显示，奥希替尼明显优于化疗，ORR分别为71%和31%（OR=5.39; 95%CI: 3.47-8.48; *P* < 0.001），PFS分别为10.1个月和4.4个月（HR=0.32; 95%CI: 0.21-0.49）^[8]^。所有预定义的亚组中均可见获益于奥希替尼的PFS。与培美曲塞+铂类组相比，奥希替尼组的患者生活质量更好。前临床数据^[9]^显示，通过在猴脑中更高的暴露量来看，奥希替尼与Rociletinib和吉非替尼相比，改善了血脑屏障的通透性。在对AURA和AURA 2患者的合并分析中，有50例患者可评估中枢神经系统反应，结果颅内ORR为54%，DCR为92%^[10]^。在AURA 3研究中，预先指定的亚组分析报告指出，奥希替尼与化疗相比具有更高的中枢神经系统（central nervous system, CNS）应答率（70% *vs* 31%）、更持久的应答（8.9个月*vs* 5.7个月）和更长的CNS PFS（11.7个月*vs* 5.6个月）^[11]^。奥希替尼的颅内疗效可能为治疗具有挑战性的*EGFR*突变患者的管理提供一种新的治疗选择^[12]^。

由于奥希替尼是*EGFR*敏感突变和T790M、野生型EGFR的选择性抑制剂，因此在AURA研究中，该药的耐受性良好。最常见的副作用是腹泻（33%-47%）、皮疹（34%-43%）、甲沟炎（17%-26%）和皮肤干燥（20%-30%），且低于第一或第二代EGFR-TKIs的研究报道^[13]^。在1%-4%的AURA研究中发现了任意等级的间质性肺疾病，同时在2%-4%的患者中报告了QTc间期延长，但Ⅲ级以上不良事件少见。

## 3 EGFR T790M的检测方式

第三代EGFR-TKIs的开发和奥希替尼被用于一线EGFR-TKIs治疗后进展的T790M阳性晚期NSCLC患者的批准为检测T790M提供了动力。对EGFR-TKI获得性耐药患者，检测T790M的标准方法是肿瘤二次活检^[14]^，患者或家属拒绝、肿瘤位置、分子检测样本不足以及患者状态都会限制其实施^[15]^。此外，同一患者存在的肿瘤内异质性和时空变异性也可能导致误诊^[16]^。

利用血浆循环肿瘤DNA（circulating tumor DNA, ctDNA）检测T790M状态是一种已成功应用于突变型患者的策略，并且具有非有创性的优点，同时可以进行动态监测^[17]^。然而，如果血浆T790M状态为阴性，若可行仍建议肿瘤组织二次活检^[18]^。

以Cobas组织检测为参照标准，评价了Cobas *EGFR*突变检测血浆T790M的性能特点。在AURA扩展试验和AURA 2的研究分析中，Cobas血浆检测血浆T790M的敏感性、特异性和总体一致性分别为61%、79%和65%。血浆和肿瘤T790M阳性患者的ORR分别为64%和66%^[19]^。在AURA 3研究中，Cobas血浆检测血浆T790M的敏感性和特异性分别为51%和77%。在血浆与组织T790M阳性的患者中，ORR（77% *vs* 71%）与PFS（8.2个月*vs* 10.1个月）相似^[20]^。在Rociletinib的Ⅰ期研究中，Cobas血浆检测的敏感性、特异性和一致性分别为64%、69%和86%^[21]^。

除血浆检测以外，实时PCR技术如Bio-Rad Droplet Digital PCR、PANAMutyper、RGQ PCR试剂盒等均可作为T790M突变的检测方法。其中Therascreen EGFR血浆RGQ PCR试剂盒用于检测EGFR外显子19、外显子20 T790M和外显子21 L858R的缺失情况，并对突变情况进行定性评价。PANAMutyper EGFR试剂盒基于肽核酸介导的实时夹闭和熔融峰分析，设计了检测47种不同EGFR变异体的方法。用PNA构建PCR钳制反应，钳制抑制野生型DNA的扩增，增加突变序列的优先扩增。而Bio-Rad Droplet Digital PCR建立了一套数字化PCR系统，对qPCR进行了优化^[13]^。

通过对Cobas、Therascreen、ddPCR^TM^和BEAMing四个平台的交叉对比研究，发现T790M检测的敏感性分别为41%、29%、71%和71%，特异性分别为100%、100%、83%和67%^[22]^。近年来，Cobas^®^ *EGFR*突变检测与MiSeq NGS的敏感性、特异性和一致性相比分别为93%、92%和92%^[19]^。

## 4 第三代EGFR-TKI的获得性耐药机制

与使用第一代或第二代EGFR-TKIs治疗的患者相似，使用奥希替尼治疗的患者最终会产生耐药性。在前临床和临床研究中都报道了几种依赖EGFR和非依赖EGFR的耐药机制（图 1）。

**1:**
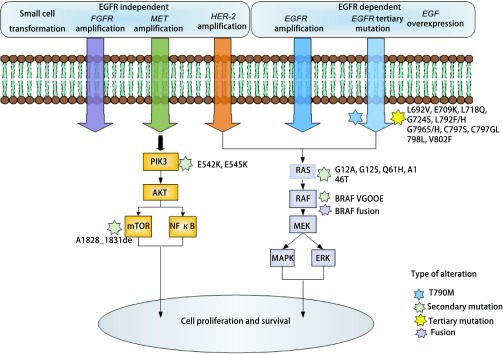
第三代EGFR-TKIs患者EGFR信号转导及EGFR依赖和独立耐药机制示意图。耐药机制报道临床样本包括*EGFR* C797S以及其他罕见的三级*EGFR*突变，*MET*、*HER-2*、*FGFR*和*KRAS*扩增、*PIK3CA*和*BRAF* V600E突变、EGF的过度表达、MAPK的激活、小细胞肺癌转化，还报道了多种复杂的分子畸变。 A simplified represented of the mechanisms of resistance to EGFR-TKIs. Reported drug-resist mechanism clinical samples include EGFR C797S and other rare tertiary *EGFR* mutations, *MET*, *HER-2*, *FGFR* and *KRAS* amplification, *PIK3CA* and *BRAF* V600E mutations, overexpression of EGF, activation of MAPK, transformation of small cell lung cancer, and a variety of complex molecular aberrations were also reported. EGFR-TKIs: epidermal growth factor receptor-tyrosine kinase inhibitors; HER-2: human epidermal growth factor receptor 2; FGFR: fibroblast growth factor receptor; KRAS: Kirsten rat sarcoma viral oncogene homolog; PIK3CA: phosphatidylin-ositol 3-kinase catalytic alpha polypeptide gene; BRAF: v-raf murine sarcoma viral oncogene homolog B1; MAPK: mitogen-activated protein kinase; NF-*κ*B: nuclear factor kappa B.

### 4.1 依赖于EGFR的机制

依赖EGFR的机制包括*EGFR* C797S突变的发生，这是第三代EGFR-TKIs最常见的耐药机制之一^[23, 24]^。这个耐药性突变是奥希替尼针对ATP结合处的半胱氨酸-797残基为靶点^[25]^，该突变发生在位于激酶结合位点的EGFR C797密码子上，导致奥希替尼与EGFR的共价结合缺失。除了奥希替尼外，在使用Rociletinib、奥莫替尼和Narzatinib治疗的EGFR T790M患者中也有C797S的报道^[26-28]^。有研究^[29]^显示，获得C797S的等位基因环境可以预测EGFR-TKI治疗的敏感性。体外研究已经报道了C797S和T790M的反式突变对第三代EGFR-TKIs有耐药性，但对第一代和第三代TKIs的联合应用敏感，而如果C797S和T790M发生顺式突变，则EGFR-TKIs单独或联合作用无效。这些前临床的发现得到了最近一份涉及一个*EGFR* T790M和C797S反式突变病例报道的支持，该病人对厄洛替尼和奥希替尼的组合有反应^[30]^。最近又报道了一种新的伴有*MYC*和*EGFR*扩增的*EGFR* C797G顺式突变^[31]^。T790M缺失引起的耐药性在早期的奥希替尼应用中经常出现，与存在T790M的患者相比，T790M缺失的患者治疗失败时间更短^[32]^。

Piotrowska等^[33]^最近的一项研究对Guardant Health数据库的61例C797S突变肺腺癌患者血浆样本的进行了评估。所有患者均有T790M突变，随后使用奥希替尼进行治疗。所观察的C797S构型示：有50例（82%）顺式C797S/T790M患者；反式C797S/T790M患者占6例（10%）；仅有C797S无T790M患者占4例（6%）；1例（2%）患者同时存在2个C797S克隆（1个T790M顺式克隆，1个反式克隆）。此外，51例（84%）患者至少有一种耐药机制与C797S同时发生；EGFR扩增（*n*=29; 48%）；*MET*扩增（*n*=10; 16%）；*BRAF* V600E（*n*=3; 5%）和*PIK3CA*突变（*n*=9; 15%）。因此，C797S的多克隆性以及真实的耐药机制反映了耐药*EGFR*突变肿瘤的异质性。

除了C797S，还有其他的罕见三级*EGFR*突变，包括新的溶剂前突变（G796S/R）、位于792位的亮氨酸残基铰链口袋突变（L792F/H）、位于798位的结合干扰（L798I）以及位于718位的空间位阻（L718Q）^[27, 34-37]^。所有的L792突变都是T790M的顺式突变和C797的反式突变。Ou等^[37]^报道，10例L792阳性患者中有2例、7例L718阳性患者中有6例没有同时存在C797突变。耐药突变的多样性可能是由于肿瘤的异质性，这代表一个治疗的挑战并对未来的药物开发有指导性作用。

由*EGFR*敏感活化突变、T790M和三级*EGFR*突变组成的三重突变是一个重要的临床挑战，第四代*EGFR*选择性突变、变构性非ATP竞争性抑制剂（如EAI045）正在开发中^[25]^。在L858R/T790M/C797S小鼠模型中，EAI045与西妥昔单抗具有协同作用。化合物Go6976的结构研究^[38]^表明，一种有效的蛋白激酶C（protein kinase C, PKC）抑制剂可优先与EGFR T790M/C797S结合，也可能克服C797S的存在。

### 4.2 非依赖EGFR的机制

在肿瘤和/或血浆样本中报道的不依赖EGFR耐药机制包括*MET*扩增^[39-41]^、*HER-2*^[39, 42]^和*FGFR*^[43]^、*MAPK*激活^[43]^、*KRAS*突变、*PI3KCA*^[35]^、*BRAF*^[44]^、*PTEN*缺失^[36]^和向小细胞肺癌（small cell lung cancer, SCLC）转化^[45]^。此外，SFK/FAK信号也可通过维持AKT和MAPK通路减弱奥希替尼在耐药模型中的疗效^[46]^。

*MET*和*HER2*扩增在临床前期和临床研究中均有报道。值得注意的是，*HER*扩增在使用奥希替尼进展的患者中似乎是相互独立的^[39-42]^，而*MET*扩增则不是^[33]^。EGFR-TKI耐药可观察到*MET*扩增，MET和EGFR双抑制可抑制小鼠模型的肿瘤生长^[41, 47]^。据报道，使用奥希替尼进展的患者存在成纤维细胞生长因子受体1（fibroblast growth factor receptor 1, *FGFR1*）扩增^[43]^。患者肿瘤组织中成纤维细胞生长因子2（fibroblast growth factor 2, FGF2）mRNA水平大约高出20倍，表明存在FGF2-FGFR1自分泌环介导的耐药。另一方面，*PIK3CA*突变（E545K）已被报道为奥希替尼的耐药机制^[48, 49]^，这个热点突变是否对PI3K抑制剂敏感还有待进一步研究。同时发生的*PIK3CA*、*PTEN*和*TP53*等突变也见于小细胞转化患者^[50]^。转化为SCLC的肿瘤可能保留原有的*EGFR*激活突变，但未发现T790M。已知视网膜母细胞瘤基因（retinoblastoma 1, RB1）的缺失也与SCLC的转化有关^[12, 43]^。

RAS-MAPK通路激活与*KRAS*突变、*KRAS*扩增、*BRAF*、*NRAS*和*MEK1*突变相关，以上均被报道为奥希替尼获得性耐药机制^[35, 42, 48, 49, 51]^。*KRAS*突变包括G12S、G12A、Q61H、G12D和A146T位点。在临床前期模型中，奥希替尼可识别*NRAS*突变（E63K）或NRAS拷贝数增加^[51]^。值得注意的是，耐药细胞系对MEK抑制剂、司美替尼和EGFR-TKI的组合表现出敏感性。也有报道^[44, 49]^认为*BRAF*突变（V600E）为奥希替尼的耐药机制之一。恩考芬尼（BRAF抑制剂）和奥希替尼的组合对耐药细胞系表现出敏感性^[44]^。

在组织学改变方面，在EGFR-TKIs治疗期间或之后，一部分患者（5%-10%）发生了NSCLC向SCLC的组织学转化^[52]^。转化为SCLC的机制基础在很大程度上仍未明确，但可能与RB缺失有关^[53]^。RB缺乏本身在SCLC中很常见，初步认为NSCLC中EGFR的慢性抑制导致了SCLC的遗传、组织学和药物敏感性的表现。小细胞转化也与上皮-间质转化（epithelial-to-mesenchymal transition, EMT）程序相关，E-cadherin低表达和波形蛋白表达升高就是证明^[52]^。EMT也可能是由AXL激酶^[54]^、TGF或Notch-1的激活引起的^[55, 56]^。转化为SCLC的肿瘤可能保留原有的*EGFR*激活突变，但未发现T790M。同时发生的*PIK3CA*、*PTEN*和*TP53*等突变也见于小细胞转化患者^[57]^。

## 5 未来的视角和总结

随着EGFR靶向治疗药物的不断增加，治疗*EGFR*突变型晚期NSCLC的最佳顺序成为了一个重要问题。在T790M突变患者中，奥希替尼优于化疗。在最近发表的FLAURA试验中，奥希替尼与吉非替尼（NCT02296125）的Ⅲ期研究中阐明与第一代EGFR-TKI相比，奥希替尼显示出更好的PFS和更少的毒性。此外，它也被证明对CNS转移患者有效^[58]^。2019年8月31日奥希替尼获批为一线治疗*EGFR*敏感性突变。T790M耐药突变的检测方式以组织学为金标准，由于客观原因无法获得组织学标本可以用血浆检测进行尝试，血浆ctDNA检测的出现和临床验证为追踪患者肿瘤的动态基因组演变提供了可能。血液检测在某些方面解决了部分患者的肿瘤异质性问题，但是如何提高检测的敏感性和特异性仍是目前存在的挑战。三代EGFR-TKI的获得性耐药机制主要包括两个方面：依赖于EGFR的机制和非依赖机制。依赖于EGFR的机制最主要的是*EGFR* C797S突变的发生，顺式突变和反式突变是C797S与T790M突变共同存在的两种形式。非依赖机制包括*MET*扩增、*HER-2*和*FGFR*、*MAPK*激活、*KRAS*突变、*PI3KCA*、*BRAF*、*PTEN*缺失和SCLC转化等。三代EGFR-TKI耐药后精准的检测技术是寻找临床应对策略的基础。

未来十年的热点将继续集中于第三代EGFR-TKIs耐药机制研究，这将对临床全程管理NSCLC起到重要作用，并对开发新一代治疗和合理联合用药策略以克服EGFR-TKI耐药起到至关重要的作用和理论依据。

## References

[1] Rosell R, Carcereny E, Gervais R (2012). Erlotinib versus standard chemotherapy as first-line treatment for European patients with advanced *EGFR* mutation-positive non-small-cell lung cancer (EURTAC): a multicentre, open-label, randomised phase 3 trial. Lancet Oncol.

[2] Neel DS, Bivona TG (2017). Resistance is futile: overcoming resistance to targeted therapies in lung adenocarcinoma. NPJ Precis Oncol.

[3] Tan CS, Cho BC, Soo RA (2016). Next-generation epidermal growth factor receptor tyrosine kinase inhibitors in epidermal growth factor receptor-mutant non-small cell lung cancer. Lung Cancer.

[4] Cross DA, Ashton SE, Ghiorghiu S (2014). AZD9291, an irreversible EGFR-TKI, overcomes T790M-mediated resistance to EGFR inhibitors in lung cancer. Cancer Discov.

[5] Janne PA, Yang JC, Kim DW (2015). AZD9291 in EGFR inhibitor-resistant non-small-cell lung cancer. N Engl J Med.

[6] Yang JC, Ahn MJ, Kim DW (2017). Osimertinib in pretreated T790M-positive advanced non-small-cell lung cancer: AURA study phase Ⅱ extension component. J Clin Oncol.

[7] Goss G, Tsai CM, Shepherd FA (2016). Osimertinib for pretreated EGFR Thr790Met-positive advanced non-small-cell lung cancer (AURA2): a multicentre, open-label, single-arm, phase 2 study. Lancet Oncol.

[8] Mok TS, Wu YL, Ahn MJ (2018). Osimertinib or platinum-pemetrexed in EGFR T790M-positive lung cancer. Lung Cancer.

[9] Ballard P, Yates JW, Yang Z (2016). Preclinical comparison of osimertinib with other EGFR-TKIs in EGFR-mutant NSCLC brain metastases models, and early evidence of clinical brain metastases activity. Clin Cancer Res.

[10] Goss G, Shepherd F, Ahn MJ (2018). CNS response to osimertinib in patients with T790M-positive advanced NSCLC: pooled data from two phase Ⅱ trials. Ann Oncol.

[11] Mok TS, Han JY, Kang JH (2018). CNS response to osimertinib in patients (pts) with T790M-positive advanced NSCLC: Data from a randomized phase Ⅲ trial (AURA3). J Clin Oncol.

[12] Tan CS, Cho BC, Soo RA (2017). Treatment options for EGFR mutant NSCLC with CNS involvement - Can patients BLOOM with the use of next generation EGFR-TKIs?. Lung Cancer.

[13] Chen YL, Lin CC, Yang SC (2019). *EGFR* five technologies for detecting the T790M mutation in the circulating cell-free DNA of patients with non-small cell lung cancer: a comparison. Front Oncol.

[14] Yoon HJ, Lee HY, Lee KS (2012). Repeat biopsy for mutational analysis of non-small cell lung cancers resistant to previous chemotherapy: adequacy and complications. Radiology.

[15] Chouaid C, Dujon C, Do P (2014). Feasibility and clinical impact of re-biopsy in advanced non small-cell lung cancer: a prospective multicenter study in a real-world setting (GFPC study 12-01). Lung Cancer.

[16] de Bruin EC, McGranahan N, Swanton C (2015). Analysis of intratumor heterogeneity unravels lung cancer evolution. Mol Cell Oncol.

[17] Sundaresan TK, Sequist LV, Heymach JV (2016). Detection of T790M, the acquired resistance EGFR mutation, by tumor biopsy versus noninvasive blood-based analyses. Clin Cancer Res.

[18] Novello S, Barlesi F, Califano R (2016). Metastatic non-small-cell lung cancer: ESMO Clinical Practice Guidelines for diagnosis, treatment and follow-up. Ann Oncol.

[19] Jenkins S, J CHY, Ramalingam SS (2017). Plasma ctDNA analysis for detection of the *EGFR* T790M mutation in patients with advanced non-small cell lung cancer. J Thorac Oncol.

[20] Wu YL, Jenkins S, Ramalingam S (2017). MA08.03 Osimertinib *vs* platinum-pemetrexed for T790M-mutation positive advanced NSCLC (AURA3): plama ctDNA analysis. J Thorac Oncol.

[21] Karlovich C, Goldman JW, Sun JM (2016). Assessment of EGFR mutation status in matched plasma and tumor tissue of NSCLC patients from a phase Ⅰ study of rociletinib (CO-1686). Clin Cancer Res.

[22] Thress KS, Brant R, Carr TH (2015). EGFR mutation detection in ctDNA from NSCLC patient plasma: A cross-platform comparison of leading technologies to support the clinical development of AZD9291. Lung Cancer.

[23] Thress KS, Paweletz CP, Felip E (2015). Acquired EGFR C797S mutation mediates resistance to AZD9291 in non-small cell lung cancer harboring EGFR T790M. Nat Med.

[24] Yu HA, Tian SK, Drilon AE (2015). Acquired resistance of EGFR-mutant lung cancer to a T790M-specific EGFR inhibitor: emergence of a third mutation (C797S) in the EGFR tyrosine kinase domain. JAMA Oncol.

[25] Jia Y, Yun CH, Park E (2016). Overcoming EGFR (T790M) and EGFR (C797S) resistance with mutant-selective allosteric inhibitors. Nature.

[26] Song HN, Jung KS, Yoo KH (2016). Acquired C797S mutation upon treatment with a T790M-specific third-generation EGFR inhibitor (HM61713) in non-small cell lung cancer. J Thorac Oncol.

[27] Chabon JJ, Simmons AD, Lovejoy AF (2016). Circulating tumour DNA profiling reveals heterogeneity of EGFR inhibitor resistance mechanisms in lung cancer patients. Nat Commun.

[28] Daniel SW, Tan D, Natasha B (2017). Genomic profiling of resistant tumor samples following progression on EGF816, a third generation, mutant-selective EGFR tyrosine kinase inhibitor, in advanced non-smal cell lung cancer. 2017 ASCO Annual Meeting. J Clin Oncol.

[29] Niederst MJ, Hu H, Mulvey HE (2015). The allelic context of the C797S mutation acquired upon treatment with third-generation EGFR inhibitors impacts sensitivity to subsequent treatment strategies. Clin Cancer Res.

[30] Wang Z, Yang JJ, Huang J (2017). Lung adenocarcinoma harboring EGFR T790M and in trans C797S responds to combination therapy of first- and third-generation EGFR-TKIs and shifts allelic configuration at resistance. J Thorac Oncol.

[31] Menon R, Muller J, Schneider P (2016). A novel EGFR (C797) variant detected in a pleural biopsy specimen from an osimertinib-treated patient using a comprehensive hybrid capture-based next-generation sequencing assay. J Thorac Oncol.

[32] Geoffrey R, Oxnard YH, Kathryn F (2017). Osimertinib resistance mediated by loss of EGFR T790M is associated with early resistance and competing resistance mechanisms. World Conference on Lung Cancer.

[33] Piotrowska Z, Fairclough S, Lanman RB (2017). Characterizing the genomic landscape of EGFR C797S in lung cancer using ctDNA next-generation sequencing. IASLC World Conference on Lung Cancer.

[34] Bersanelli M, Minari R, Bordi P (2016). L718Q mutation as new mechanism of acquired resistance to AZD9291 in EGFR-mutated NSCLC. J Thorac Oncol.

[35] Chen K, Zhou F, Shen W (2017). Novel mutations on EGFR Leu792 potentially correlate to acquired resistance to osimertinib in advanced NSCLC. J Thorac Oncol.

[36] Yang Z, Yang N, Ou Q (2018). Investigating novel resistance mechanisms to third-generation EGFR tyrosine kinase inhibitor osimertinib in non-small cell lung cancer patients. Clin Cancer Res.

[37] Ou SI, Cui J, Schrock AB (2019). Emergence of novel and dominant acquired *EGFR* solvent-front mutations at Gly796 (G796S/R) together with C797S/R and L792F/H mutations in one EGFR (L858R/T790M) NSCLC patient who progressed on osimertinib. Lung Cancer.

[38] Kong LL, Ma R, Yao MY (2017). Structural pharmacological studies on EGFR T790M/C797S. Biochem Biophys Res Commun.

[39] Planchard D, Loriot Y, Andre F (2015). EGFR-independent mechanisms of acquired resistance to AZD9291 in EGFR T790M-positive NSCLC patients. Ann Oncol.

[40] Ou SI, Agarwal N, Ali SM (2016). High *MET* amplification level as a resistance mechanism to osimertinib (AZD9291) in a patient that symptomatically responded to crizotinib treatment post-osimertinib progression. Lung Cancer.

[41] Shi P, Oh YT, Zhang G (2016). *Met* gene amplification and protein hyperactivation is a mechanism of resistance to both first and third generation EGFR inhibitors in lung cancer treatment. Cancer Lett.

[42] Ortiz-Cuaran S, Scheffler M, Plenker D (2016). Heterogeneous mechanisms of primary and acquired resistance to third-generation EGFR inhibitors. Clin Cancer Res.

[43] Kim TM, Song A, Kim DW (2015). Mechanisms of acquired resistance to AZD9291: a mutation-selective, irreversible EGFR inhibitor. J Thorac Oncol.

[44] Ho CC, Liao WY, Lin CA (2017). Acquired *BRAF* V600E mutation as resistant mechanism after treatment with osimertinib. J Thorac Oncol.

[45] Ham JS, Kim S, Kim HK (2016). Two cases of small cell lung cancer transformation from EGFR mutant adenocarcinoma during AZD9291 treatment. J Thorac Oncol.

[46] Ichihara E, Westover D, Meador CB (2017). SFK/FAK signaling attenuates osimertinib efficacy in both drug-sensitive and drug-resistant models of EGFR-mutant lung cancer. Cancer Res.

[47] Martinez-Marti A, Felip E, Matito J (2017). Dual MET and ERBB inhibition overcomes intratumor plasticity in simertinib-resistant-advanced non-small-cell lung cancer (NSCLC). Ann Oncol.

[48] Ramalingam SS, Yang JC, Lee CK (2018). Osimertinib as first-line treatment of *EGFR* mutation-positive advanced non-small-cell lung cancer. J Clin Oncol.

[b49] 49Oxnard GR. Mechanisms of acquired resistance to AZD9291 in EGFR T790 M positive lung cancer. IASLC 16th World Conf Lung Cancer, Denver, Colorado, 2015

[50] Li L, Wang H, Li C (2017). Transformation to small-cell carcinoma as an acquired resistance mechanism to AZD9291: A case report. Oncotarget.

[51] Eberlein CA, Stetson D, Markovets AA (2015). Acquired resistance to the mutant-selective EGFR inhibitor AZD9291 is associated with increased dependence on RAS signaling in preclinical models. Cancer Res.

[52] Sequist LV, Waltman BA, Dias-Santagata D (2011). Genotypic and histological evolution of lung cancers acquiring resistance to EGFR inhibitors. Sci Transl Med.

[53] Niederst MJ, Sequist LV, Poirier JT (2015). RB loss in resistant EGFR mutant lung adenocarcinomas that transform to small-cell lung cancer. Nat Commun.

[54] Zhang Z, Lee JC, Lin L (2012). Activation of the AXL kinase causes resistance to EGFR-targeted therapy in lung cancer. Nat Genet.

[55] Capaccione KM, Hong X, Morgan KM (2014). Sox9 mediates Notch1-induced mesenchymal features in lung adenocarcinoma. Oncotarget.

[56] Yao Z, Fenoglio S, Gao DC (2010). TGF-beta IL-6 axis mediates selective and adaptive mechanisms of resistance to molecular targeted therapy in lung cancer. Proc Natl Acad Sci U S A.

[57] Li L, Wang H, Li C (2017). Transformation to small-cell carcinoma as an acquired resistance mechanism to AZD9291: A case report. Oncotarget.

[58] Soria JC, Ohe Y, Vansteenkiste J (2018). Osimertinib in untreated EGFR-mutated advanced non-small-cell lung cancer. N Engl J Med.

